# Resuscitation of Pulsed Electric Field-Treated *Staphylococcus aureus* and *Pseudomonas putida* in a Rich Nutrient Medium

**DOI:** 10.3390/foods10030660

**Published:** 2021-03-19

**Authors:** Efrat Emanuel, Irina Dubrovin, Roman Pogreb, Gad A. Pinhasi, Rivka Cahan

**Affiliations:** 1 Department of Chemical Engineering, Ariel University, Ariel 40700, Israel; efiem80@gmail.com (E.E.); Irinadu@ariel.ac.il (I.D.); gadip@ariel.ac.il (G.A.P.); 2 Department of Physics, Ariel University, Ariel 40700, Israel; ptoman@ariel.ac.il

**Keywords:** pulsed electric fields, current density, conductivity, eradication, bacteria

## Abstract

Pulsed electric fields (PEFs) technology was reported to be useful as a disinfection method in the liquid food industry. This technology may lead to membrane permeabilization and bacterial death. However, resuscitation of viable but non-culturable cells and sublethally injured microorganisms in food was reported to be associated with foodborne outbreaks. The main aim of this study was to investigate the possible recovery of injured PEF-treated bacteria. The PEF treatment of *Staphylococcus aureus* and *Pseudomonas putida* led to a reduction of 3.2 log_10_ and 4.8 log_10_, respectively. After 5 h, no colony forming units (CFUs) were observed when the bacteria were suspended in phosphate buffer saline (PBS); and for 24 h, no recovery was observed. The PEF-treated *S. aureus* in brain-heart infusion (BHI) medium were maintained at 1.84 × 10^4^ CFU mL^−1^ for about 1.5 h. While *P. putida* decreased to zero CFU mL^−1^ by the 4th hour. However, after that, both bacteria recovered and began to multiply. Flow cytometry analysis showed that PEF treatment led to significant membrane permeabilization. Mass spectrometry analysis of PEF-treated *P. putida* which were suspended in BHI revealed over-expression of 22 proteins, where 55% were related to stress conditions. Understanding the recovery conditions of PEF-treated bacteria is particularly important in food industry pasteurization. To our knowledge, this is the first comprehensive study describing the recovery of injured PEF-treated *S. aureus* and *P. putida* bacteria.

## 1. Introduction

Applying a threshold external pulsed electric field (PEF) to cells leads to an increase in their membrane permeability, a phenomenon which is termed electroporation. Based on experiments and theoretical studies, it was proposed that this phenomenon occurs when the external electric field exceeds the capacity of the cell membrane potential, leading to mechanical changes and a rapid breakdown in the cell membrane. Hydrophilic pores are created when water molecules enter through the membrane lipid bilayer, causing the polar head groups of adjacent phospholipids to face toward the water [1,2,3]. There are four electric field ranges characterized by their membrane electroporation properties: No detectable electroporation, reversible electroporation, non-thermal irreversible electroporation, and irreversible electroporation with thermal damage [4]. The electric field ranges depend on the cell type and size, the medium components, its osmolarity, and electrical conductivity [5,6]. Below a specific field strength, regardless of the duration of the applied electric field, there is no detectable electroporation [7,8]. The reversible electroporation range is characterized by pore formation providing the transport of molecules. However, sometimes after applying the electric pulse, there is a pore resealing, where most of the electroporated cells retain their viability. In the nonthermal irreversible electroporation range, the pores reseal too slowly or not at all, causing release of the cell contents. In the fourth range, the irreversible electroporation with thermal damage, the electric current increases the temperature, leading to a denaturation of the cellular molecules [2]. The pore formation was reported to occur in less than a second, while resealing occurred over a range of minutes [9,10].

In the context of resealing injured cells, bacterial viability is commonly evaluated based on its ability to replicate, and the lack of replication is an indicator of nonexistence of microbial life. However, active replication depends on different environmental and stress conditions [11,12]. For microorganisms, the designation of live or dead is not clear. Yet neither the process from cell life to death nor the reverse route of recovery are fully understood. Viable but non-culturable (VBNC) cells, as well as sublethally injured microorganisms, are important forms of life that may be induced by stress conditions, which include disinfection methods such as heat treatment, hydrostatic pressure, ultraviolet radiation, pulsed light, cold plasma, and PEF treatment [13,14].

It is important to differentiate between the VBNC state of cells and sublethally injured cells. The VBNC state may be defined as an inactive form of life due to stress conditions [14]. VBNC cells maintain membrane integrity and possess low metabolic activity as well as gene expression; but the formation of colony forming units (CFUs) is inhibited. Suitable environmental conditions may lead to a recovery of VBNC cells [15]. Resuscitation describes the VBNC cells’ recovery process from the nonculturable to the culturable state. This process is triggered by the increase or decrease of the temperature, increased nutrient concentration, or induced chemical stimuli [16,17]. In contrast to VBNC cells, sublethally injured cells can still multiply, but they do so very slowly and on nonselective growth media. Sublethal injury is induced by exposure to physical or chemical processes. Under suitable conditions, a repair process may occur, which leads to regrowth [11,18].

The resuscitation of VBNC cells in food may take place during shelf-life storage [19]. It was reported that foodborne outbreaks could be associated with VBNC states [20].

PEF technology was reported to be useful as a disinfection method in water purification processes and the liquid food industry. PEF by nanowire-assisted low-voltage proved effective for bacterial inactivation with low energy cost [21]. This technology is considered a “clean” method not accompanied by heating; it therefore does not change food flavor, color, and taste [22,23,24]. A reduction of microorganisms was previously reported in PEF processing of nectar [25], milk [26], liquid eggs [27], and wine [28]. The extent of bacterial disinfection using PEF depends on the treatment chamber configuration, the bacterial cell type, the medium, and the applied electric parameters [29,30,31]. The electric field strength necessary for bacterial disinfection was reported to be as high as 100 kV cm^−1^ or as low as 1–4.5 kV cm^−1^. However, most of the reported electric fields are in tens of kV cm^−1^ [31,32,33,34]. The applied pulse duration ranges from ns to ms with an exponential or square pattern [25,35], and the pulse number is between one and tens of thousands [36,37].

This study aimed to investigate the possible recovery of VBNC or/and sublethally injured PEF-treated *S. aureus* (Gram-positive) and *P. putida* F1 (Gram-negative) bacteria when the cells are suspended in a rich nutrient medium compared to phosphate buffer saline (PBS). The bacterial suspension in PBS was treated with a moderate pulsed electric field (2.9 kV cm^−1^, 3.4 A cm^−2^), followed by dilution in PBS and a nutrient-rich brain heart infusion (BHI) medium. The replication ability of the bacteria was measured by the quantity of CFUs. The membrane permeability and cell size were measured using flow cytometry (FCM) analysis. In addition, the proteome of the recovered bacteria was compared to that of the nontreated cells by mass spectrometry (MS) analysis. To our knowledge, this is the first comprehensive study describing the recovery of injured bacteria in a rich medium. Describing the recovery phenomenon of injured PEF-treated cells, and thereby understanding their recovery conditions, is particularly important in food industry sterilization. 

## 2. Materials and Methods

### 2.1. Growth Conditions

*S. aureus* (25923) was purchased from ATCC (Manassas, VA, USA) and *P. putida* F1 (6899) from DSMZ (Braunschweig, Germany). The bacteria were grown in BHI agar for 24 h at 37 °C. A few isolated colonies were suspended overnight in 12.5 mL mineral medium (MM) supplied with 1% glucose as the carbon source [38], followed by incubation at 25 °C and low agitation at 70 rpm, in order to reach the log phase at the beginning of the experiment. The culture was centrifuged (Avanti J-E centrifuge, Beckman Coulter, Brea, CA, USA) at 4000 g for 10 min at 4 °C, and the sediment was washed with ultrapure (UP) water with a resistance of 18.4 MΩ-cm at 25 °C (Synergy UV water purification system, Merck, Darmstadt, Germany). The bacterial sediment was suspended in different concentrations of PBS and UP water, to a final optical density of 0.01 at 600 nm, using a spectrophotometer (Genesys 10S UV-VIS, Thermo Scientific, Waltham, MA, USA).

### 2.2. PBS Solution

10 mM PBS (1.47 mM KH_2_PO_4_, 8.1 mM Na_2_HPO_4_, 2.67 mM KCl, 136.9 mM NaCl) was purchased from Biological Industries (Beit HaEmek, Israel). Different PBS concentrations (0.089–0.54 mM) were prepared by diluting in UP water.

### 2.3. Solution pH and Conductivity

The pH of the UP water and PBS were 4.8 ± 0.2 and 4.5–5.2 ± 0.2, respectively. The conductivity of the UP water and PBS were 1 and 155–1050 µS cm^−1^, respectively. The conductivity was measured before adding the bacteria, using a conductivity meter (4168, Traceable^®^ Products, Webster, TX, USA).

### 2.4. Total Specific Energy

The total specific energy ($W_{T}$) was calculated as described in the work of Raso et al. (2016) [39]. Calculation of the specific energy input per pulse (W) is shown in Equation (1):

(1) $$W=\frac{1}{m}\int_{0}^{\infty }U\left(t\right)\cdot I\left(t\right)dt\;\;\;$$

The *W* is the integral over time of the recorded pulse shape of current and voltage that was measured on the treatment chamber during the pulse (τ) where m is the sample mass, *U(t)* is the voltage, and *I(t)* is the current measured on the PEF chamber during load pulse (τ). The total specific energy ($W_{T}$) was determined by multiplying the specific energy per pulse (*W*) with the pulse number (*n*), shown in Equation (2):

(2) $$W_{T}=W\cdot n$$

In this research, the number of pulses were 5000, but the energy per pulse changed in correlation to the current that was generated in line with the culture’s conductivity.

### 2.5. Heat Transfer Model

The temperature response in the system was calculated using COMSOL Multiphysics numerical software [40] (COMSOL Multiphysics, http://www.comsol.com, accessed on 5 February 2021). The 3D transient heat transfer model was based on the conduction heat transfer in the sample and the electrodes’ domain, and on the heat convection at the electrodes’ boundaries. The heat generation source term was taken from the total specific energy calculations. The initial temperature of the entire system (sample and electrodes) was 22 °C. The heat generated during the system operation was transferred to the electrodes and their surroundings. The heat transfer model was based on the conduction heat equation for each domain (electrodes and samples), seen in Equation (3):

(3) $$\frac{1}{\propto_{i}}\frac{\partial T}{\partial t}=\nabla^{2}T+\frac{q^{\prime \prime \prime }}{k_{i}}$$

where *T* is the temperature in the space (x, y, z) and time (*t*), *T* (x y z, t), *q’’’* is the heat source (W m^−3^), $\alpha_{i}$ is the heat diffusivity, and $k_{i}$ is the heat conductivity (i = e for the electrodes, or *s* for the sample). As a boundary condition, we took the convection heat transfer at the electrode walls.

Equation (4):

(4) $$-k_{e}{\frac{\partial T}{\partial x}|}_{w}=h{\left(T_{w}-T_{\infty }\right)}_{\;}$$

where h is the convection heat coefficient and $T_{\infty }$ is the ambient temperature.

### 2.6. Design and Construction of the Electroporator

The design and construction of the electroporator was done according to our previous study [35]. In brief, a high-voltage generator (900 V) was used to apply an electric field to the bacterial suspension. The voltage pulses were controlled by a synthesized function generator. The voltage between the electrodes of the chamber and the current was controlled using a two-channel oscilloscope. The current density was calculated in accordance with cross-section S (J_CH_ = I_CH_/S; Supplementary material Figure S1).

#### 2.6.1. Construction of the Electroporator Chamber

**Figure 1:**
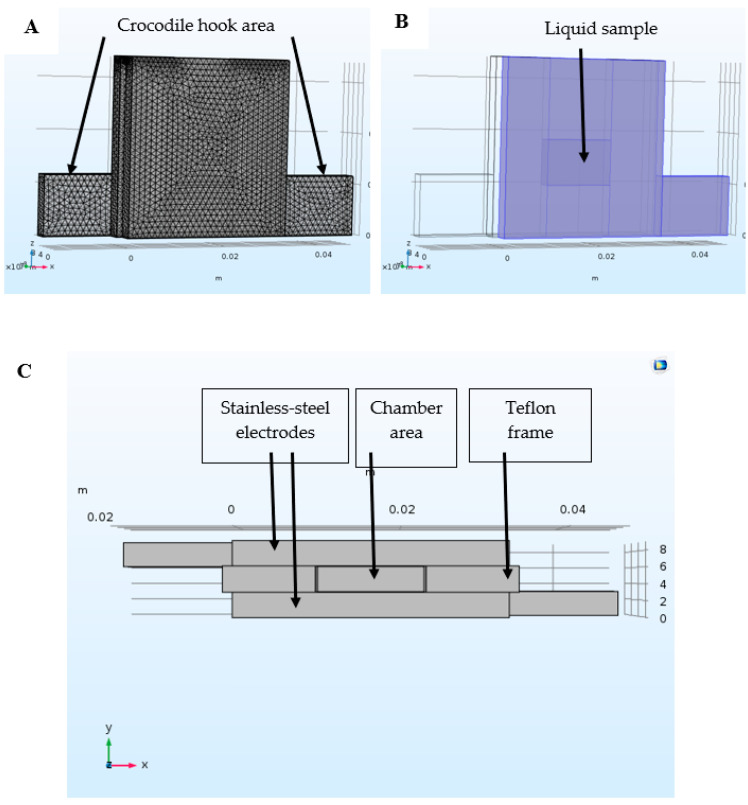
Schematic drawing of the electroporator chamber: Side view (**A**,**B**); top view (**C**).

The electroporate chamber (Figure 1) was made from two stainless-steel plates (thickness of 3 mm, a width of 32.7 mm, and height of 33.94 mm). The lower part of each electrode (14.64 mm width × 11.6 mm height) was used for attaching the crocodile hook. A special clamp was used to tightly press the electrodes with a Teflon frame. The chamber gap was 3.1 mm, width 13.1 mm and height 24.26 mm; it was filled up to a height of 8.7 mm with only 350 µL suspension (the current cross-section S = 1.13 cm^2^).

#### 2.6.2. Applied PEF Characterization

The bacterial suspensions were exposed to an electric field of 2.9 kV cm^−1^ with a frequency of 100 Hz. Each pulse duration was 10 µs with a square shape. The pulses were performed in a continuous series of 10 trains (500 pulses in each train). The train duration was 5 s with a 2 s interval between the trains. The chamber voltage U_CH_ polarity was alternated for each train [34,41].

#### 2.6.3. PEF Procedure for Determining the Conditions of Bacterial Eradication

The bacteria were suspended in PBS which was diluted in UP water (0–0.54 mM PBS), leading to current densities of between 0.02 ± 0.01–3.4 ± 0.1 A cm^−2^. The bacterial sample was treated by pulsed electric-field conditions as described above. The initial temperature was 22 °C in all experiments. The temperature of the bacterial suspension at the end of the PEF treatment was measured with a multimeter (VICHY, VC99) type-K (chromel-alumel) thermocouple; temperatures did not exceed 35 °C. Immediately after PEF treatment, the bacterial suspension was transferred to an Eppendorf tube and incubated at 37 °C for 2 h, followed by CFU analysis.

#### 2.6.4. PEF Procedure for Determining Membrane Permeability, Bacterial Size and Viability as a Function of Dilution in PBS and BHI Medium

The bacterial suspension (350 µL) in 0.54 mM PBS was exposed to electric-field conditions as described above. This experiment was performed twice, and the suspensions were mixed to achieve a volume of 700 µL. The bacterial suspension was then divided into three parts. One portion was examined immediately after the PEF treatment by viable count assay and FCM analysis. A second portion was diluted in BHI medium, and the third portion in 0.54 mM PBS; both at a ratio of 1:10 and incubated at 37 °C for 24 h. At indicated times, the cultures were sampled for CFU count and FCM analysis. The same procedure was done for the control samples but without exposure to PEF treatment.

### 2.7. Viable Count Assay

The PEF-treated suspension (100 µL) was diluted and the appropriate dilutions were pour-plated onto BHI agar plates, followed by incubation for 24 h at 37 °C. Viable cells were determined by CFU count, which were multiplied by the appropriate dilutions. The same procedure was performed for the nontreated culture (control). 

### 2.8. Examination of Bacterial Membrane Permeability and Cell Size by FCM Analysis

The PEF-treated bacterial suspension and the nontreated bacteria were diluted 10-fold in 0.54 mM PBS and BHI, as detailed above. This was followed by adding fluorescent propidium iodide (PI) at a final concentration of 1.5 µM. The samples (about 50,000 cells) were incubated for 5 min at 37 °C and passed through a CytoFLEX flow cytometer (Beckman Coulter, Atlanta, GA, USA). Data were analyzed using FlowJo software (Tree Star, San Carlos, CA, USA).

### 2.9. Proteolysis and Mass Spectrometry Analysis

The pulsed electric field-treated bacterial suspension and nontreated bacteria were diluted in BHI medium at a ratio of 1:10 and incubated at 37 °C for 6 h. The cultures were collected and centrifuged at 4000× *g* for 10 min at 4 °C (Avanti J-E centrifuge, Beckman Coulter, Atlanta, CA, USA). The sediment was washed three times with 1 mL PBS and the sediment of the fourth centrifuge was stored at −80 °C.

#### 2.9.1. Proteolysis

The proteins were extracted from the PEF-treated and nontreated (control) pellets via two cycles of sonication in 10 mM DTT, 400 mM ammonium bicarbonate and 9 M urea. 7 µg protein from each sample were treated with 35.2 mM iodoacetamide in 400 mM ammonium bicarbonate (30 min in the dark at room temperature) and digested in 1 M urea and 44 mM ammonium bicarbonate, with modified trypsin (Promega) at a 1:50 enzyme-to-substrate ratio (overnight at 37 °C). Then, a second trypsinization (4 h) was performed.

#### 2.9.2. Mass Spectrometry Analysis

The tryptic peptides were desalted using C18 tips, then dried and resuspended in 2% acetonitrile and 0.1% formic acid. The peptides were resolved by reverse-phase chromatography on 0.075 × 180-mm fused silica capillaries (J&W) packed with Reprosil reversed phase material (Dr Maisch GmbH, Germany). The peptides were eluted with a linear 180 min gradient of 5 to 28%, 15 min with a gradient of 28 to 95%, and 25 min in 95% acetonitrile with 0.1% formic acid in water at flow rates of 0.15 μlmin^−1^. Mass spectrometry was done as described by Michael et al., 2017 [42]. The mass spectrometry data from three biological repeats were analyzed using MaxQuant software 1.5.2.8 (Mathias Mann’s Group) vs. the *Pseudomonas putida* proteome (strain ATCC 700007/DSM 6899/BCRC 17059/F1) from the Uniprot database (Proteome ID: UP000000556, 5245 entries), with 1% FDR (false discovery rate). The data was quantified by label-free analysis using the same software, based on extracted ion currents (XICs) of peptides, enabling quantitation from each LC/MS run for each peptide identified in any of the experiments.

### 2.10. Statistics

Data were expressed as means ± SE (standard error) of between three to five replicates. The paired *t-*test was used for estimation of statistical significance. The results were considered statistically significant at *p* ˂ 0.05.

## 3. Results and Discussion

### 3.1. Total Specific Energy and the Temperature Profile

To rule out the possibility of a thermal effect on the results, energy balance and heat transfer analysis were performed to find the temperature distribution during the PEF treatment operation. The measurements were performed for the highest conductive sample with 1050 µS cm^−1^ (the worst-case scenario). The electrode potential and current were measured overtime during the operation. The measured potential and current are presented in Supplementary Material Figure S2.

The total specific energy measurement was obtained from numerical integration using the potential and current data during the duration of pulses per sample mass, as described in Section 2.4. For the experiments where maximum current was applied, the total specific energy was found to be 640 kJ kg^−1^. This value was used as the heat source for the heat transfer modeling.

The temperature profile along the system for different times is presented in Supplementary Material Figure S3A, and the center maximum-temperature time history is presented in Supplementary Material Figure S3B. The temperature map after 70 s is presented in Supplementary Material Figure S3C.

It emerged that after operation time of 70 s, the average temperature in the sample was approximately 35 °C. The temperature predictions (see Supplementary Material Figure S3B) were consistent with the experimental results measured in the sample temperature history.

The relatively low temperature obtained at the end of the process was consistent with the assumption that the disinfecting effect of the system is under the influence of the electric field and is not due to thermal effects. The total specific energy for the other samples were approximated by using the experimental results for the voltage and current response for the known tested sample (with the highest conductivity), and the conductivity of each sample (Ohm’s law).

### 3.2. PEF Conditions for P. putida F1 and S. aureus Eradication

In this study, the PEF conditions for bacterial eradication were examined for *S. aureus* and *P. putida* F1, which served as models of Gram-positive and Gram-negative bacteria, respectively. These bacteria are known to contaminant different kinds of foods. *P. putida* metabolic activity in aerobically stored meat leads to volatile compounds production, which affects meat spoilage [43]. *S. aureus* is also known as a foodborne pathogen that contaminates meat products [44].

**Figure 2:**
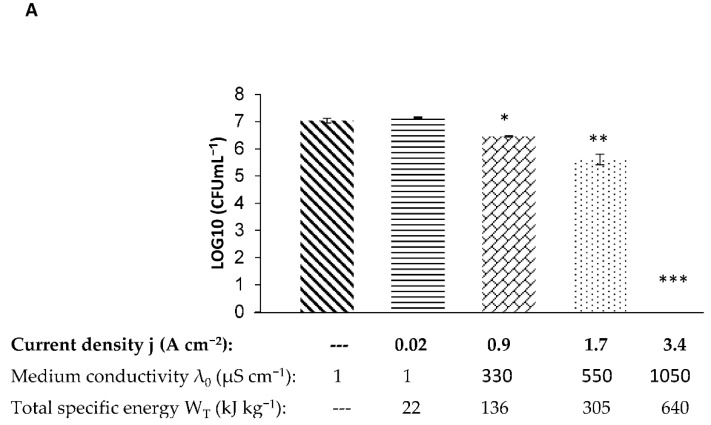

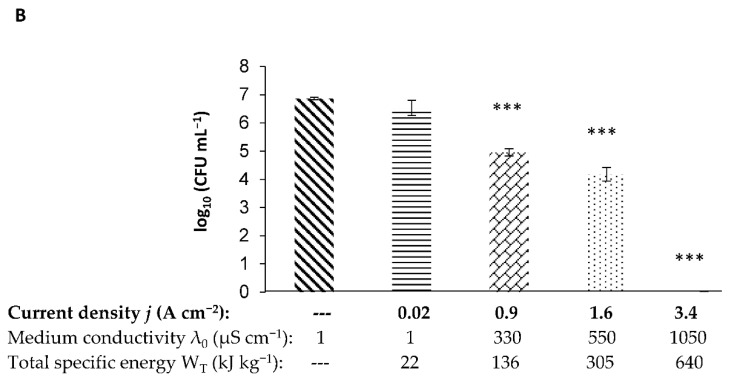
CFU (colony forming unit) counts of *S. aureus* (**A**) and *P. putida* F1 (**B**) as a function of pulsed electric field (PEF)-treatment in different current densities. The control column and the second column (0.02 A cm^−2^) represent the CFU mL^−1^ of bacterial cells that were suspended in UP water without PEF-treatment and with PEF-treatment, respectively. The remaining columns (0.9–3.4 A cm^−2^) represent the CFU mL^−1^ that were suspended in solutions with different current densities. *p*-value (*t*-test): *p* < 0.05 *; *p* < 0.01 **; *p* < 0.001 ***.

The bacteria in their log phase were suspended in PBS that was previously diluted in UP water (0–0.54 mM) to achieve a conductivity range of 1–1050 µS cm^−1^ and a final optical density of 0.01 at 600 nm. The bacterial suspensions were exposed to an electric field of 2.9 kV cm^−1^, 100 Hz, square pulse shape with a duration of 10 µs. The pulses numbered 5000 in a train mode. The current densities were between 0.02 ± 0.01 and 3.4 ± 0.1 A cm^−2^ as described for each sample. The PEF-treated bacteria were incubated for 2 h at 37 °C, and the surviving bacteria were examined by a viable count assay (Figure 2).

The result of exposure of *S. aureus* to an electric field of 2.9 kV cm^−1^ in different current densities is shown in Figure 2A. As can be seen, bacterial death was found to be dependent on the current density. No eradication was observed at a current density of 0.02 ± 0.01 A cm^−2^. At current densities of 0.9 ± 0.1 and 1.7 ± 0.1 A cm^−2^, the CFU mL^−1^ decreased by 0.6 and 1.4 log_10_, respectively. Total bacterial eradication was observed at a current density of 3.4 ± 0.1 A cm^−2^ (7.0 log_10_ reduction). For *P. putida* F1 (Figure 2B), a decrease in bacterial viability of 0.3, 1.9, and 2.7 log_10_ CFU mL^−1^ was observed at current densities of 0.02 ± 0.01, 0.9 ± 0.1, and 1.6 ± 0.1 A cm^−2^, respectively. Total bacterial eradication was observed at a current density of 3.4 ± 0.1 A cm^−2^ (6.9 log_10_ reduction).

In summary, when *S. aureus* and *P. putida* F1 were suspended in UP water and exposed to PEF intensity of 2.9 kV cm^−1^, the reduction of CFU mL^−1^ was not significant. However, when the bacterial samples were suspended in increasing PBS concentrations (leading to an increase of buffer conductivity as well as current density), total eradication of *S. aureus* (7.0 log_10_ reduction) and of *P. putida* F1 (6.9 log_10_ reduction) was observed at a conductivity of 1050 µS cm^−1^ and current density of 3.4 ± 0.1 A cm^−2^.

The effect of the current density in PEF treatment of 4, 2.8, 2, and 1 kV cm^−1^ on *P. putida* F1 eradication was comprehensively examined in our previous study. In each of the tested electric-field strengths, increased current density led to higher bacterial death. In an electric field of 4 kV cm^−1^, total bacterial eradication was observed at a current density of 2 ± 0.2 A cm^−2^. In a lower electric field of 1 kV cm^−1^, total bacterial eradication was observed at a higher current density of 5.2 ± 0.5 A cm^−2^. The conclusion from these results is that in a certain electric field, increasing the current density may lead to a higher bacterial eradication [34].

However, the effect of current density on bacterial eradication is the subject of controversy. It was reported that a similar electric-field strength was needed for *Saccharomyces cerevisiae* suspended in orange juice and skim milk, having a conductivity of 3.7 mS cm^−1^ and 4.5 mS cm^−1^, respectively. However, a higher strength of electric-field was needed in a sodium alginate solution with a conductivity of 5.4 mS cm^−1^ [41]. Jayaram et al. showed that bacterial eradication in a higher conductive solution (2230 µS cm^−1^) was less effective, compared to a lower conductivity of 170 µS cm^−1^ [45].

However, a phenomenon similar to our results, namely a linear correlation between increasing cell-suspension conductivity and bacterial eradication, was observed by other researchers. Siemer et al. (2013) examined bacterial endospore inactivation using PEF treatment when combined with thermal energy. They reported a reduction of 2.5 log in *B. subtilis* spores in a solution with a conductivity of 4000 µS cm^−1^ when exposed to an electric-field strength of 9 kV cm^−1^; and only 4 kV cm^−1^ was needed in a higher conductive solution of 15,000 µS cm^−1^ [46]. Pucihar et al. (2001) found a linear correlation between the increase in medium conductivity (0.001, 0.005, 0.14, and 1.6 S m^−1^) and the percentage of cell death [5].

Many studies have focused on the influence of PEF parameters on bacterial cell eradication, such as electric-field strength, total specific energy, pulse number and shape, medium sort, and conductivity. However, the influence of the current density on bacterial eradication was barely investigated. We assume that cell damage may also be influenced by the current density, which is correlated to the charged particle concentration and type.

It was reported that monovalent or divalent cations may react differently with the membrane phospholipid headgroups, with the consequence of different levels of damage to the cell membranes [47]. The bonds between the ions and membrane phospholipids can change the moment dipole and net tilt of headgroups. In addition, the ion’s charge and size can influence the direction and magnitude of these shifts [48]. Suspension of microorganisms in equally conductive solutions but with different ion compositions (NaCl, MgCl_2_, CaCl_2_, MgSO_4,_ or KCl) showed variations in extent of the permeabilization with NaCl, leading to larger membrane permeabilization compared to the other ions [49].

In conclusion, we have found a linear correlation between increases in the current density and bacterial eradication. We assume that both electric-field strength and medium conductivity have an influence on bacterial eradication.

### 3.3. Viability of PEF-Treated Bacteria as a Function of Suspension in BHI Medium and PBS

In this experiment, the bacteria were exposed to PEF-treatment (2.9 kV cm^−1^ at current density of 3.4 ± 0.1 A cm^−2^, frequency of 100 Hz) with a pulse duration of 10 µs and 5000 pulses in a train mode of 500 pulses each (with alternating polarity for each train). The CFU count was examined during 24 h.

**Figure 3:**
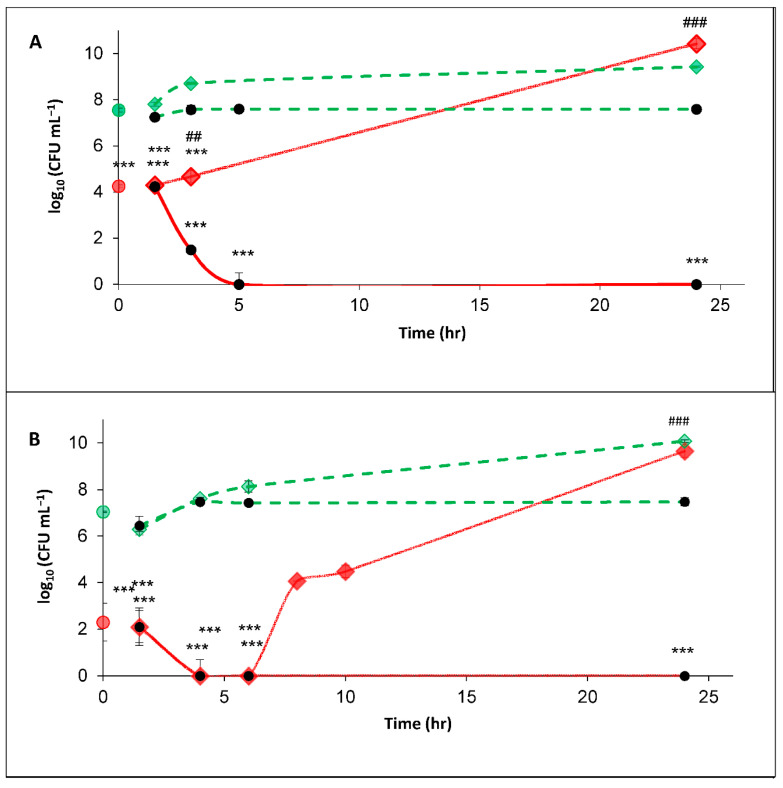
CFU mL^−1^ of PEF-treated and nontreated *S. aureus* (**A**) and *P. putida* F1 (**B**). PEF-treated bacteria in time ‘0’ (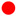); non-treated bacteria in time ‘0’ (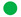); PEF-treated bacteria in brain-heart infusion (BHI) (1.5–24 h) (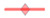); PEF-treated bacteria in phosphate buffer saline (PBS) (1.5–24 h) (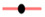); nontreated bacteria in BHI (1.5–24 h) (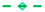); non-treated bacteria in PBS (1.5–24 h) (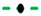). *p* value (t test): Significance of the CFU count in each examined time (PEF-treated bacteria in BHI or in BPS) related to its control (nontreated bacteria in BHI or PBS, respectively) *p* < 0.001 ***; significance of the CFU of the treated-bacteria in PBS related to treated-bacteria in BHI, in each examined time *p* < 0.01 ##; *p* < 0.001 ###.

The PEF-treated bacterial suspension (0.02 OD 600 nm) was divided into three portions. The first portion (100 µL) was examined immediately after the PEF-treatment for CFU mL^−1^ (time ‘0’). The second portion (100 µL) was suspended in 900 µL of 0.54 mM PBS and designated as PEF-treated bacteria in PBS, while the third (100 µL) was diluted in 900 µL BHI and designated as PEF-treated bacteria in BHI (in order to imitate liquid in the food industry). The same procedure was performed on bacterial suspensions that were not exposed to PEF-treatment, designated as nontreated bacteria in BHI and nontreated bacteria in PBS. The PEF-treated and nontreated bacteria were incubated for 24 h at 37 °C, and at indicated times, a viable count assay was performed (Figure 3).

The CFU count of the PEF-treated *S. aureus* bacteria at time ‘0’ was 1.84 × 10^4^, while the nontreated bacteria exhibited 3.78 × 10^7^ CFU mL^−1^. That is, the PEF-treatment led to a reduction of 3.2 log_10_. The nontreated bacteria in PBS remained in about the same concentration during the entire experiment; at 24 h the count was 3.93 × 10^7^ CFU mL^−1^. The nontreated bacteria in BHI continued to replicate, and after 24 h, it reached 3.40 × 10^10^ CFU mL^−1^. The CFU counts of the PEF-treated *S. aureus* in PBS after 1.5 and 3 h were 1.92 × 10^4^ and 7.50 × 10^1^ CFU mL^−1^, respectively. Meanwhile, after 5 h and until the end of the experiment, no CFUs were observed (Figure 3A). A different result was obtained for the PEF-treated bacteria in BHI. After 3 h, there were 6.97 × 10^4^ CFU mL^−1^ which continued to replicate, and at 24 h reached 2.73 × 10^10^ CFU mL^−1^, about the same as the nontreated bacteria in BHI.

The phenomenon of *P. putida* F1 survival was slightly different (Figure 3B). The CFU count of the PEF-treated bacteria at time ‘0’ was 1.97 × 10^2^ CFU mL^−1^, while the nontreated bacteria exhibited 1.13 × 10^7^ CFU mL^−1^. That is, the PEF-treatment led to a reduction of 4.8 log_10_. The nontreated bacteria in PBS remained in the same concentration during the entire experiment, and the nontreated bacteria in BHI continued to replicate, reaching 1.20 × 10^10^ CFU mL^−1^ after 24 h. However, no CFUs of the PEF-treated bacteria in PBS or BHI were observed from the 4th to the 6th hours after treatment. The PEF-treated bacteria in BHI began to replicate after the 6th hour, by the 8th hour reaching 1.18 × 10^4^ CFU mL^−1^. At the end of the experiment (24 h), the CFU counts were similar to those in the BHI control (the nontreated bacteria).

It was previously stated that the thick peptidoglycan layer and structural properties of the Gram-positive bacterial membrane protect them from PEF damage. For this reason, Gram-positive bacteria are more resistant than Gram-negative to PEF treatment [3,50]. García et al. (2005) reported on different sensitivities of Gram-positive and Gram-negative bacteria to PEF-treatment, which was correlated to the pH of the medium. The Gram-negative bacteria *Escherichia coli* O157:H7 exhibited higher resistance to PEF treatment at pH 4, while Gram-positive *Listeria monocytogenes* bacteria exhibited higher resistance to PEF treatment at pH 7 [51].

Other studies showed the effectiveness of PEF-treatment in decreasing S. aureus and Pseudomonas in the milk industry. Cregenzán-Alberti et al., 2015, showed a reduction of PEF-treated S. aureus in milk by 5.2 log_10_ at 32.5 °C, 40 kV cm^−1^ and 89 μs, and a decrease by 5.3 log_10_ of Pseudomonas fluorescens at 32.5 °C at slightly higher PEF (42.5 kV cm^−1^) and a longer treatment time (106 μs) [52]. Pankaj-Sharma et al., 2014, examined the inactivation of Gram-positive and Gram-negative bacteria in whole milk using PEF-treatment combined with pre-heating. The milk was exposed to 18–28 kV cm^−1^ for 17–235 μs at different temperatures for 24 s. At 4 °C, PEF-treatment did not affect the bacterial CFU. However, the increasing temperature increased the PEF-treatment effectiveness. PEF-treatments at 22–28 kV cm^−1^ for 17–101 μs at 50 °C led to a 5–6 log reduction Pseudomonas aeruginosa, and Staphylococcus aureus were reduced to below the detection limit at 55 °C [53]. Craven et al., 2008, showed that PEF-treatment of milk led to increasing the shelf-life of fresh milk. This was examined by the time taken to reach 10^7^ CFU mL^− 1^ of Pseudomonas. It was reported that the greatest inactivation (>5 logs) was achieved at 55 °C with 31 kV cm^−1^ (139.4 kJ L^−1^). Heat treatment at this temperature without PEF-treatment caused a reduction of Pseudomonas by only 0.2 logs. PEF-treatment of inoculated milk by Pseudomonas (10^3^ and 10^5^ CFU mL^−1^), extended the shelf-life by at least 8 days at 4 °C compared with nontreated milk. From these results, it can be evaluated that PEF-treatment is a useful method for reducing Pseudomonas, the major spoilage bacteria of milk [54].

The phenomenon of VBNC cells which was induced by the PEF-treatment, was reported to be caused by other conventional disinfection methods. For example, *E. coli* and *P. aeruginosa* enter a VBNC state when exposed to UV irradiation [55], *Listeria monocytogenes* when treated by high temperature (60 °C, for 3–9 min) [56], *S. cerevisiae* in the presence of SO_2_ (200–250 ppm) for 12 days [57], and *E. coli* when exposed to different stress conditions such as H_2_O_2_ (0.05%), osmotic shock (13% NaCl at 37 °C), low pH (Acetic acid, pH 3.0), UV irradiation, and heat (56 °C for 6 h) [58].

In conclusion, our results demonstrated that the Gram-positive bacteria *S. aureus* were more resistant to PEF-treatment compared to the Gram-negative bacteria *P. putida*. In addition, *P. putida* F1 entered a VBNC state or sublethal injured when exposed to PEF-treatment. Since PEF becomes an important disinfection method in the liquid food industry, the PEF-injured cells should be comprehensively investigated.

### 3.4. Membrane Permeability of PEF-Treated Bacteria Suspended in BHI Medium and PBS

**Figure 4:**
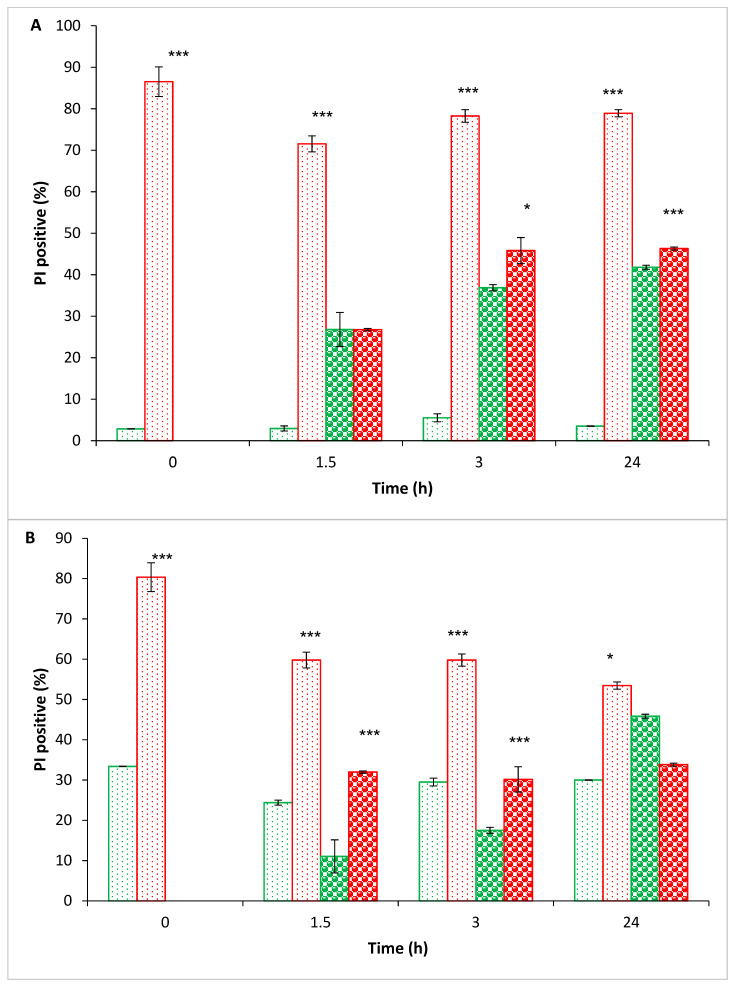
Membrane permeability of *S. aureus* (**A**) and *P. putida* F1 (**B**). PEF-treated bacteria which were diluted in BHI (1.5–24 h) (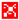); PEF-treated bacteria which were suspended in PBS (0–24 h) (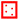); nontreated bacteria which were diluted in BHI (1.5–24 h) (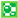); nontreated bacteria which were suspended in PBS (0–24 h) (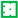). Propidium iodide (PI)-positive control using ethanol was 90 ± 3%. *p* values (*t*-test): *p* < 0.05 *; *p* < 0.001 ***.

The effect of PEF treatment on the membrane permeability of *S. aureus* and *P. putida* F1 was examined using FCM analysis. A suspension (350 µL) of PEF-treated bacteria (0.02 OD 600 nm) in 0.54 mM PBS was divided into three parts. The first portion (120 µL) was used to examine the membrane permeability in time ‘0’. The second portion (100 µL) was diluted in 0.54 mM PBS (1:10) and designated as PEF-treated bacteria in PBS. The third portion (100 µL) was diluted in BHI medium (1:10), labeled as PEF-treated bacteria in BHI. The same procedure was performed on the nontreated bacteria in 0.54 mM PBS (0.02 OD 600 nm), which were diluted in BHI and PBS and designated as nontreated bacteria in BHI and nontreated bacteria in PBS, respectively. The bacterial suspensions were incubated for 24 h at 37 °C; and at selected times (0, 1.5, 3 and 24 h), PI was added to a sample of 120 µL for 5 min, followed by examination of the membrane permeability using FCM analysis (Figure 4).

As shown in Figure 4A, the percentage of the PI-positive *S. aureus* cells (A) in time ‘0’ of the PEF-treated bacteria was 86 ± 4%, while the nontreated sample exhibited only 3 ± 0.05%. The PI-positive percentages of the nontreated bacteria in BHI after 1.5 h was 27 ± 4%, which increased to 37 ± 0.7% and 42 ± 0.5% after 3 and 24 h, respectively.

Similar results were obtained with the PEF-treated bacteria in BHI: after 1.5 h, the PI-positive percentage was 27 ± 0.3%, whereas after 3 and 24 h, the PI-positive percentage was about 46%.

The nontreated bacteria in PBS exhibited a low PI-positive percentage of about 3 ± 0.1% during the 24 h, presumably a result of the intact membrane and the inability to multiply in PBS. In contrast, the PEF-treated bacteria in PBS exhibited a PI-positive percentage of 86 ± 4% at time ‘0’, which only slightly decreased to 79 ± 0.9% over 24 h.

The PI-positive percentage of the nontreated *P. putida* F1 in PBS (Figure 4B) at time ‘0’ was 33 ± 1% and remained about the same during the entire experiment. It is important to note that the FCM analysis took into account only whole cells, and not the debris located in another area.

The PI-positive percentages of the nontreated bacteria in BHI (at 1.5, 3 and 24 h) were 11, 18, and 45%, respectively, and the PEF-treated bacteria in BHI were about 30% at any given time. We assume that the high PI-positive percentages of the PEF-treated bacteria evolved of the time for cells resealing, whereas the high PI-positive percentages of the nontreated bacteria in BHI depended on cell physiology. As was reported earlier, PI uptake in the Gram-negative *Sphingomonas* sp. LB126 and the Gram-positive *Mycobacterium frederiksbergense* was found to be dependent on their bacterial physiology. Up to 40% of both strains were stained by PI during early exponential growth on glucose, compared to 2–5% of cells in the early stationary phase of growth [59]. In addition, it was previously reported that when transferring intact untreated cells into phosphate or Tris buffers, a high percentage of cells were stained with whatever fluorescent dye is used [33].

An interesting result was observed at time ‘0’ with the PEF-treated *P. putida*, which were diluted in PBS. A high percentage (20%) of PI-negative cells were observed (Figure 4B), while Figure 3B showed a high reduction (5 log) of bacterial eradication. From the results shown in Figure 3B, less PI-negative cells would have been expected. It is important to note that PI-negative cells do not indicate that the membrane is fully intact. Given that PI is a somewhat large molecule, and it could be that a low molecular weight dye would show a higher percentage of permeable cells. It is also possible that the majority of the 20% PI-negative cells were in a stress mode that influenced their multiplication and ability to form colonies.

Another interesting phenomenon was observed with the PEF-treated *S. aureus* and *P. putida*, which were diluted in PBS. At time ‘0’, the treated bacteria exhibited PI-positive percentages of 86 ± 4% and 80 ± 2%, respectively. Over time, these percentages were reduced, and by the end of the experiment, they were down to 79 ± 0.9% (*p* value < 0.001) and 53 ± 1%, respectively. We assume that over time, some of the permeable cells were lysed and accumulated as debris. Since the FCM analysis only counts whole cells, a false result of fewer PI-positive cells was observed. This assumption may also be confirmed by the results shown in Figure 3, where the CFU counts of the PEF-treated *S. aureus* and *P. putida* in PBS were reduced to zero from after the 5th hour until the end of the experiment.

### 3.5. Cell Size of PEF-Treated Bacterial as a Function of Dilution in BHI Medium and PBS

**Figure 5:**
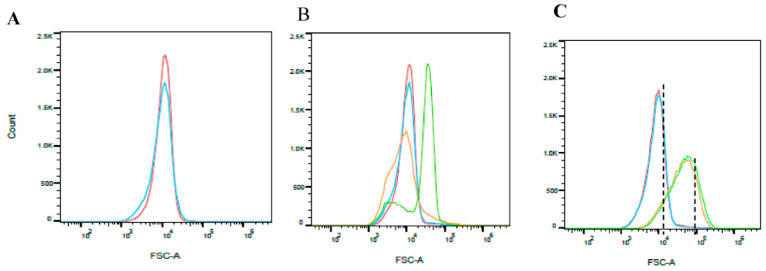
FCM (flow cytometry) analysis of *S. aureus* relative cell size. (**A**–**C**) represent comparison between the PEF-treated and nontreated samples at selected times: (**A**)—‘0’ time; (**B**)—1.5 h; (**C**)—24 h. Nontreated cells in PBS-red; nontreated cells in BHI-orange; PEF-treated cells in BHI-green; PEF-treated cells in PBS-blue.

The bacterial suspensions were exposed to an electric field of 2.9 kV cm^−1^, followed by examining the bacterial cell size using FCM analysis (Figure 5). Each examined sample included about 50,000 cells, so that the area under each curve was equal.

The relative cell size of *S. aureus* bacteria after PEF treatment is shown in Figure 5. At ‘0’ time, the nontreated *S. aureus* in PBS (red line) had a size similar to the PEF-treated *S. aureus* in PBS (blue line): Between ~2 × 10^3^ and ~3 × 10^4^, with a sharp peak in ~1_*_10^4^. This phenomenon continued until the end of the experiment at 24 h. At 1.5 h, the nontreated bacteria in BHI (orange line) exhibited a wide curve, with ~1 × 10^3^–~4 × 10^4^ representing a wide variation of cell size. The PEF-treated bacteria in BHI (green line) exhibited two different sub-populations, one of which ranged between ~2 × 10^3^–~1 × 10^4^ and the other ~2 × 10^4^–~1 × 10^5^. At 24 h, the PEF-treated *S. aureus* in BHI exhibited the same cell size as the nontreated bacteria in BHI; sizes ranged from ~4 × 10^3^ to ~3 × 10^5^, with a peak in ~6 × 10^4^.

In summary, during the experiment the relative cell size of the PEF-treated *S. aureus* in PBS exhibited the same sizes as the nontreated bacteria in PBS, without changing between time ‘0’ and 24 h. However, 1.5 h after exposure, the PEF-treated *S. aureus* in BHI exhibited two sub-populations, with one presenting a larger relative cell size compared to the nontreated sample. At 24 h, the cell size was relatively large, but the same as that of the nontreated bacteria in BHI. We assume that the large cells evolved according to membrane permeabilization, which allowed water diffusion.

The same experiment was performed with *P. putida*. However, no difference was observed here in the relative cell sizes of the treated vs. nontreated bacteria during the experiment.

It has been reported that following PEF treatment, three different microbial populations may be found: Live, dead, and sublethally injured cells where the membrane is damaged, but there is still metabolic activity [60]. The population composition may depend on the physical PEF parameters, microbial classification, and environmental conditions such as medium, pH, and temperature [3]. In the range of reversible electroporation, microorganisms with injured membranes may able to overcome the damage and recover.

This process occurs under certain conditions such as the type of medium, optimal growth pH, and temperature [61]. The recovery of sublethally injured *S. cerevisiae* cells after PEF treatment was investigated in different media and acidity conditions. Sublethally injured *S. cerevisiae* were detected after PEF treatment at 12.0 kV cm^−1^ in citrate-phosphate buffer, with a pH of 7.0 and 4.0. The recovery of the sublethally injured cells occurred when they were transferred to citrate–phosphate buffer (pH 4.0). No recovery was detected when the cells were suspended in citrate–phosphate buffer (pH 7.0) [62].

Kinosita and Tsong (1977) studied the effect of temperature on pore resealing in PEF-treated erythrocytes; they found that pore recovery was in the range of hours to days at 4 °C, while at 37 °C, it took only minutes to hours [9]. Garcia et al. (2006) described the existence of two different sub-populations of PEF-treated *E. coli*. At ambient temperature, the majority (95%) of the bacteria were able to reseal their pores within 2 h. However, less than 5% of the bacterial population resealed their pores after 2 min [63]. Arroyo et al. (2010) reported that 45% of the surviving PEF-treated *Cronobacter sakazakii* which were suspended in a buffer (pH 4) were able to heal their pores almost immediately after exposure to 25 kV cm^−1^ [64].

### 3.6. MS Analysis of the Proteins from PEF-Treated P. putida F1 Suspension, Compared to Nontreated Bacteria

PEF-treated bacteria, which were immediately diluted to 1:10 in BHI (three independent experiments), and nontreated bacteria (control), which were also diluted in BHI (three independent experiments), were examined for MS analysis. The samples were taken 6 h after the PEF treatment, followed by centrifuge and washing in PBS (×3). The proteins in the sediment were extracted and digested, as described in Materials and Methods. The MS analysis was performed at the Smoler Proteomics Center at the Technion, Israel. The proteins (2160) in the MS analysis (Supplementary Material Figure S4) were identified with at least two unique peptides and 1% FDR in one of the triplications. The proteins that were taken into account for the quantification analysis (a total of 22) were limited to those with at least two unique peptides in two of the three replicates, and those that were significantly over-expressed in the PEF-treated bacteria (*p* < 0.05). The proteins consisted of three main groups: 55% were found to be related to stress conditions (related studies are cited below), 36% to various proteins, and 9% to uncharacterized proteins (Supplementary Material Figure S4). As shown in S4, the proteins related to stress conditions constituted about 55% of the over-expressed proteins. The details of these proteins and their quantitative analysis are presented in Table 1.

As can be seen in Table 1, there were 12 proteins that were significantly increased as a consequence of PEF-treatment. Alkyl hydroperoxide reductase (A5W5H2) is an enzyme related to a large family of thiol-specific antioxidant proteins which reportedly protect bacteria from abiotic stresses [65]. The alkyl hydroperoxide reductase is a crucial enzyme for gut *Bifidobacteria*, helping to manage reactive oxygen species (ROS) effectively under conditions of oxidative stress [66].

Three types of TonB-dependent siderophore receptors were identified (A5VXD9, A5W124, and A5W341). These proteins are located in the bacteria’s outer membrane; they are known for binding and transporting ferro-chelating siderophores, vitamin B12, carbohydrates, and nickel complexes [67]. A positive correlation was reported between the expression of iron-uptake systems in *P. aeruginosa* and the response to oxidative stress [68].

The integral membrane sensor-signal transducer, histidine kinase (A5VZF9), is an enzyme found in bacteria and plays a significant role in adapting to changes in the environment. It was found to be involved in bacteriocin production, quorum sensing, stress response, and regulation of malate, as well as nitrogen metabolism and resistance to antimicrobial peptides [69].

The probable proton/glutamate-aspartate symporter (A5VWS0) aids the transport of these acidic amino acids [70]. The connection between glutamate transport and stress response was previously suggested for *Lactococcus lactis* [71].

The OmpW family (A5VXU7), part of the outer membrane protein (Omp) class, is a major component of the Gram-negative bacteria’s outer membrane. The OmpW has a broad range of physiological functions, such as bacterial tolerance to antibiotics, hypergravity, herbicides, osmotic stress, and the support of bacterial survival in harsh environmental conditions like extreme temperature. In addition, OmpW enables the transport of hypochlorous acid and hydrogen peroxides as a hydrophobic ion channel; downregulation of OmpW was found to protect bacteria against damage caused by these molecules [72,73].

The protein Propeptide PepSY and peptidase M4 (A5W1V5) are related to nutrient production and pathogenicity. PepSY domains could be important in controlling cell (or spore) envelope integrity and composition under stress conditions [74].

The two proteins, the L-glutamate ATP-binding cassette (ABC) (A5VZG2) and the ABC-type metal-ion transporter (A5W1U8), are related to a large efflux pump superfamily, the ABC transporters. Several studies showed an increase in ABC transporters as a response to osmotic stress [75,76].

Deoxyribonuclease (DNase) I (A5W312) promotes catalysis of the endonucleolytic cleavage of DNA to 5′-phosphodinucleotide and 5′-phosphooligonucleotide end-products. The major role of DNases is to protect the cell from foreign DNA attack [77].

The choline/carnitine/betaine transporter (A5WA94) is coupled to sodium or proton transporters and is known as an accumulated solutes system that ensures cell turgor at high osmolarity. Osmotically controlled uptake systems allow the scavenging of organic osmolytes (osmostress protectants) from scarce environmental sources [78].

In summary, the 12 proteins that significantly increased after PEF treatment were found to be related to different stress conditions, as described for each protein.

## 4. Conclusions

Applying a threshold external pulsed electric field to biological membranes leads to an increase in their membrane permeability and pore creation. A reversible recovery of the injured cells was reported to occur under different conditions. This study investigated the recovery of injured PEF-treated bacteria, Gram-positive *S. aureus*, and Gram-negative *P. putida* F1, when the cells are suspended in a rich nutrient medium, compared to suspension in PBS.

When *S. aureus* and *P. putida* F1 were suspended in UP water and exposed to a PEF intensity of 2.9 kV cm^−1^, the reduction of CFU mL^−1^ was not significant. However, when the bacterial samples were suspended in increasing PBS concentrations, a total eradication of *S. aureus* and *P. putida* F1 was observed at a current density of 3.4 ± 0.1 A cm^−2^.

The viability of PEF-treated bacteria as a function of suspension in BHI medium and PBS was examined during 24 h. The Gram-positive *S. aureus* bacteria were more resistant to PEF-treatment compared to the Gram-negative *P. putida* F1. No CFUs were observed in either PEF-treated bacteria in PBS.

PEF-treated *P. putida* decreased to zero CFU mL^−1^ at the 4th hour for about 2 h, and then began to multiply.

The PEF-treatment led to significant membrane permeability. However, diluting the PEF-treated bacteria in a rich BHI medium led to a recovery in membrane permeability. When the PEF-treated bacteria were diluted in PBS, no recovery in membrane permeability was observed.

MS analysis of the proteins from the PEF-treated *P. putida* F1 suspension showed over-expression of 22 proteins, compared to the nontreated bacteria. 55% of these proteins were found to be related to different stress conditions.

The results of this study show a recovery of injured PEF-treated bacteria only in the rich BHI medium. In PBS, no recovery was observed in terms of CFU count and membrane permeability.

## Figures and Tables

**Table 1:** MS (mass spectrometry) analysis of the over-expressed proteins from PEF-treated *P. putida* F1, compared to the nontreated bacteria.

Protein IDs	Protein Name	Gene Name	Mol. Weight [kDa]	log_2_ * LFQ Intensity Nontreated	log_2_ * LFQ Intensity PEF-Treated
A5W5H2	Alkyl hydroperoxide reductase/Thiol specific antioxidant/Mal allergen	Pput_3256	20.507	28.96 ± 0.43	33.33 ± 0.33
A5VXD9	TonB-dependent siderophore receptor	Pput_0376	88.459	19.18 ± 1.94	24.63 ± 1.18
A5W124	TonB-dependent siderophore receptor	Pput_1678	90.426	ND	24.12 ± 0.28
A5W341	TonB-dependent siderophore receptor	Pput_2412	79.564	ND	21.21 ± 0.73
A5VZF9	Integral membrane sensor signal transduction histidine kinase	Pput_1108	70.283	ND	21.84 ± 1.71
A5VWS0	Probable proton/glutamate-aspartate symporter	gltP	47.566	21.96 ± 0.55	25.61 ± 0.18
A5VXU7	OmpW family protein	Pput_0539	24.19	ND	26.06 ± 1.47
A5W1U8	ABC-type metal ion transport system periplasmic component/surface adhesin-like protein	Pput_1965	30.162	18.25 ± 2.17	22.68 ± 0.16
A5W1V5	Propeptide, PepSY and peptidase M4	Pput_1972	44.145	ND	24.35 ± 1.01
A5VZG2	L-glutamate ABC transporter membrane protein/L-aspartate ABC transporter membrane protein	Pput_1111	27.45	17.90 ± 1.57	22.14 ± 1.10
A5W312	Deoxyribonuclease I	Pput_2383	35.477	ND	21.05 ± 0.18
A5WA94	Choline/carnitine/betaine transporter	Pput_4934	73.501	20.91 ± 0.14	24.61 ± 0.52

Note: * Since a *t*-test must be done on normally distributed data, thus mass-spectrometry data is log-normalized, we applied log_2_ transformation to the (label-free quantitation) LFQ intensities.

## Data Availability

Data available in a publicly accessible repository.

## Supplementary Materials

The following are available online at https://www.mdpi.com/2304-8158/10/3/660/s1, Figure S1: Schematic drawing of the high voltage generator and the electronic circuit; Figure S2: Potential and current time response during a pulse for two cases of opposite polarities (**A** and **B**), Voltage input () Voltage output (), current output (). Temperature in the electroporation chamber. Figure S3: Temperature profile at different times during the PEF treatment along the electrodes (**A**); Maximum temperature () in the electrode sample system, and average temperature () in the electrode sample system (**B**); Electrode sample system’s temperature map after 70 s operation time (**C**). Figure S4: Over-expressed proteins in PEF-treated *P. putida* F1 in BHI, compared to the non-treated sample.

## References

[1] Neumann E., Kakorin S., Tœnsing K. (1999). Fundamentals of electroporative delivery of drugs and genes. Bioelectrochem. Bioenerg..

[2] Kotnik T., Frey W., Sack M., Meglič S.H., Peterka M., Miklavčič D. (2015). Electroporation-based applications in biotechnology. Trends Biotechnol..

[3] Schottroff F., Krottenthaler A., Jaeger H. (2017). Stress induction and response, inactivation, and recovery of vegetative microor-ganisms by pulsed electric fields. Handbook of Electroporation.

[4] Yarmush M.L., Golberg A., Serša G., Kotnik T., Miklavčič D. (2014). Electroporation-Based Technologies for Medicine: Principles, Applications, and Challenges. Annu. Rev. Biomed. Eng..

[5] Pucihar G., Kotnik T., Kandušer M., Miklavčič D. (2001). The influence of medium conductivity on electropermeabilization and survival of cells in vitro. Bioelectrochemistry.

[6] Baldwin W.H., Gregory B.W., Osgood C.J., Schoenbach K.H., Kolb J.F. (2010). Membrane Permeability and Cell Survival after Nanosecond Pulsed-Electric-Field Exposure—Significance of Exposure-Media Composition. IEEE Trans. Plasma Sci..

[7] Kramar P., Miklavcic D., Lebar A.M. (2007). Determination of the lipid bilayer breakdown voltage by means of linear rising signal. Bioelectrochemistry.

[8] Pucihar G., Krmelj J., Reberšek M., Napotnik T.B., Miklavčič D. (2011). Equivalent Pulse Parameters for Electroporation. IEEE Trans. Biomed. Eng..

[9] Kinosita K., Tsong T.Y. (1977). Voltage-induced pore formation and hemolysis of human erythrocytes. Biochim. Biophys. Acta BBA Biomembr..

[10] Rols M., Teissié J. (1990). Electropermeabilization of mammalian cells. Quantitative analysis of the phenomenon. Biophys. J..

[11] Espina L., García-Gonzalo D., Pagán R. (2016). Detection of Thermal Sublethal Injury in Escherichia coli via the Selective Medium Plating Technique: Mechanisms and Improvements. Front. Microbiol..

[12] Davis C. (2014). Enumeration of probiotic strains: Review of culture-dependent and alternative techniques to quantify viable bacteria. J. Microbiol. Methods.

[13] Schottroff F., Fröhling A., Zunabovic-Pichler M., Krottenthaler A., Schlüter O., Jäger H. (2018). Sublethal Injury and Viable but Non-culturable (VBNC) State in Microorganisms During Preservation of Food and Biological Materials by Non-thermal Processes. Front. Microbiol..

[14] Colwell R.R., Epstein S.S. (2009). Viable but not cultivable bacteria. Uncultivated Microorganisms.

[15] Ayrapetyan M., Oliver J.D. (2016). The viable but non-culturable state and its relevance in food safety. Curr. Opin. Food Sci..

[16] Kell D.B., Kaprelyants A.S., Weichart D.H., Harwood C.R., Barer M.R. (1998). Viability and activity in readily culturable bacteria: A review and discussion of the practical issues. Antonie Leeuwenhoek Int. J. Gen. Mol. Microbiol..

[17] Bogosian G., Bourneuf E.V. (2001). A matter of bacterial life and death. EMBO Rep..

[18] Li L., Mendis N., Trigui H., Oliver J.D., Faucher S.P. (2014). The importance of the viable but non-culturable state in human bacterial pathogens. Front. Microbiol..

[19] Koukkidis G., Haigh R., Allcock N., Jordan S., Freestone P. (2016). Salad Leaf Juices Enhance Salmonella Growth, Colonization of Fresh Produce, and Virulence. Appl. Environ. Microbiol..

[20] Ferro S., Amorico T., Deo P. (2018). Role of food sanitising treatments in inducing the ‘viable but nonculturable’ state of microorganisms. Food Control..

[21] Huo Z.-Y., Zhou J.-F., Wu Y., Wu Y.-H., Liu H., Liu N., Hu H.-Y., Xie X. (2018). A Cu3P nanowire enabling high-efficiency, reliable, and energy-efficient low-voltage electroporation-inactivation of pathogens in water. J. Mater. Chem. A.

[22] Jaeger H., Schulz M., Lu P., Knorr D. (2012). Adjustment of milling, mash electroporation and pressing for the development of a PEF assisted juice production in industrial scale. Innov. Food Sci. Emerg. Technol..

[23] Saldaña G., Puértolas E., Álvarez I., Meneses N., Knorr D., Raso J. (2010). Evaluation of a static treatment chamber to investigate kinetics of microbial inactivation by pulsed electric fields at different temperatures at quasi-isothermal conditions. J. Food Eng..

[24] Knorr D., Froehling A., Jaeger H., Reineke K., Schlueter O., Schoessler K. (2011). Emerging Technologies in Food Processing. Annu. Rev. Food Sci. Technol..

[25] Evrendilek G.A., Altuntas J., Sangun M.K., Zhang H.Q. (2013). Apricot Nectar Processing by Pulsed Electric Fields. Int. J. Food Prop..

[26] Sobrino-López A., Martin-Belloso O. (2009). Review: Potential of High-Intensity Pulsed Electric Field Technology for Milk Processing. Food Eng. Rev..

[27] Amiali M., Ngadi M.O., Smith J.P., Raghavan V.G.S. (2006). Inactivation of Escherichia coli O157:H7 and Salmonella enteritidis in Liquid Egg White Using Pulsed Electric Field. J. Food Sci..

[28] Puértolas E., López N., Condón S., Raso J., Álvarez I. (2009). Pulsed electric fields inactivation of wine spoilage yeast and bacteria. Int. J. Food Microbiol..

[29] Neumann E., Schaefer-Ridder M., Wang Y., Hofschneider P. (1982). Gene transfer into mouse lyoma cells by electroporation in high electric fields. EMBO J..

[30] Ereineke K., Eschottroff F., Emeneses N., Eknorr D. (2015). Sterilization of liquid foods by pulsed electric fieldsâ€“an innovative ultra-high temperature process. Front. Microbiol..

[31] Alkhafaji S.R., Farid M. (2007). An investigation on pulsed electric fields technology using new treatment chamber design. Innov. Food Sci. Emerg. Technol..

[32] Guionet A., David F., Zaepffel C., Coustets M., Helmi K., Cheype C., Packan D., Garnier J.-P., Blanckaert V., Teissié J.E. (2015). coli electroeradication on a closed loop circuit by using milli-, micro- and nanosecond pulsed electric fields: Comparison between energy costs. Bioelectrochemistry.

[33] Coustets M., Ganeva V., Galutzov B., Teissie J. (2015). Millisecond duration pulses for flow-through electro-induced protein extraction from E. coli and associated eradication. Bioelectrochemistry.

[34] Emanuel E., Roman P., Cahan R. (2019). Influence of the current density in moderate pulsed electric fields on P. putida F1 eradication. Bioelectrochemistry.

[35] Emanuel E., Dubrovin I., Hanya E., Pinhasi G.A., Pogreb R., Cahan R. (2020). Eradication of *Saccharomyces cerevisiae* by Pulsed Electric Field Treatments. Microorganisms.

[36] French D.M., Uhler M.D., Gilgenbach R.M., Lau Y.Y. (2009). Conductive versus capacitive coupling for cell electroporation with nanosecond pulses. J. Appl. Phys..

[37] Khan S.I., Blumrosen G., Vecchio D., Golberg A., Mccormack M.C., Yarmush M.L., Hamblin M.R., Austen W.G. (2016). Eradication of multidrug-resistant pseudomonas biofilm with pulsed electric fields. Biotechnol. Bioeng..

[38] Cahan R., Stein M., Anker Y., Langzam Y., Nitzan Y. (2013). Innovative utilization of coal bottom ash for bioremediation of toxic organic pollutants. Int. Biodeterior. Biodegrad..

[39] Raso J., Frey W., Ferrari G., Pataro G., Knorr D., Teissie J., Miklavčič D. (2016). Recommendations guidelines on the key information to be reported in studies of application of PEF technology in food and biotechnological processes. Innov. Food Sci. Emerg. Technol..

[40] COMSOL Multiphysics, Documentation for COMSOL Release 5.4 2019. https://www.comsol.com/.

[41] Grahl T., Markl H. (1996). Killing of microorganisms by pulsed electric fields. Appl. Microbiol. Biotechnol..

[42] Michael E., Gomila M., Lalucat J., Nitzan Y., Pechatnikov I., Cahan R. (2017). Proteomic Assessment of the Expression of Genes Related to Toluene Catabolism and Porin Synthesis inPseudomonas stutzeriST-9. J. Proteome Res..

[43] Papadopoulou O.S., Iliopoulos V., Mallouchos A., Panagou E.Z., Chorianopoulos N., Tassou C.C., Nychas G.-J.E. (2020). Spoilage Potential of Pseudomonas (P. fragi, P. putida) and LAB (Leuconostoc mesenteroides, Lactobacillus sakei) Strains and Their Volatilome Profile during Storage of Sterile Pork Meat Using GC/MS and Data Analytics. Foods.

[44] Patarata L., Novais M., Fraqueza M.J., Silva J.A. (2020). Influence of Meat Spoilage Microbiota Initial Load on the Growth and Survival of Three Pathogens on a Naturally Fermented Sausage. Foods.

[45] Jayaram S., Castle G., Margaritis A. (1993). The effects of high field DC pulse and liquid medium conductivity on survivability of Lactobacillus brevis. Appl. Microbiol. Biotechnol..

[46] Siemer C., Toepfl S., Heinz V. (2014). Inactivation of Bacillus subtilis spores by pulsed electric fields (PEF) in combination with thermal energy—I. Influence of process- and product parameters. Food Control..

[47] Böckmann R.A., Grubmüller H. (2004). Multistep Binding of Divalent Cations to Phospholipid Bilayers: A Molecular Dynamics Study. Angew. Chem. Int. Ed..

[48] Sachs J.N., Nanda H., Petrache H.I., Woolf T.B. (2004). Changes in Phosphatidylcholine Headgroup Tilt and Water Order Induced by Monovalent Salts: Molecular Dynamics Simulations. Biophys. J..

[49] Muraji M., Tatebe W., Berg H. (1998). The influence of extracellular alkali and alkaline-earth ions on electropermeation of Saccharomyces cerevisiae. Bioelectrochem. Bioenerg..

[50] Hülsheger H., Potel J., Niemann E.G. (1983). Electric field effects on bacteria and yeast cells. Radiat. Environ. Biophys..

[51] García D., Gomez N., Raso J., Pagán R. (2005). Bacterial resistance after pulsed electric fields depending on the treatment medium pH. Innov. Food Sci. Emerg. Technol..

[52] Cregenzán-Alberti O., Halpin R., Whyte P., Lyng J., Noci F. (2015). Study of the suitability of the central composite design to predict the inactivation kinetics by pulsed electric fields (PEF) in Escherichia coli, Staphylococcus aureus and Pseudomonas fluorescens in milk. Food Bioprod. Process..

[53] Sharma P., Bremer P., Oey I., Everett D. (2014). Bacterial inactivation in whole milk using pulsed electric field processing. Int. Dairy J..

[54] Craven H., Swiergon P., Ng S., Midgely J., Versteeg C., Coventry M., Wan J. (2008). Evaluation of pulsed electric field and minimal heat treatments for inactivation of pseudomonads and enhancement of milk shelf-life. Innov. Food Sci. Emerg. Technol..

[55] Zhang S., Ye C., Lin H., Lv L., Yu X. (2015). UV Disinfection induces a VBNC state in Escherichia coli and Pseudomonas aerugino-sa. Environ. Sci. Technol..

[56] Lavieri N.A., Sebranek J.G., Cordray J.C., Dickson J.S., Jung S., Manu D.K., Mendonça A.F., Brehm-Stecher B.F., Stock J., Stalder K.J. (2014). Evaluation of the Thin Agar Layer Method for the Recovery of Pressure-Injured and Heat-Injured Listeria monocytogenes. J. Food Prot..

[57] Divol B., Lonvaud-Funel A. (2005). Evidence for viable but nonculturable yeasts in botrytis-affected wine. J. Appl. Microbiol..

[58] Asakura H., Igimi S., Kawamoto K., Yamamoto S., Makino S.-I. (2005). Role of in vivo passage on the environmental adaptation of enterohemorrhagicEscherichia coliO157:H7: Cross-induction of the viable but nonculturable state by osmotic and oxidative stresses. FEMS Microbiol. Lett..

[59] Shi L., Günther S., Hübschmann T., Wick L.Y., Harms H., Müller S. (2007). Limits of propidium iodide as a cell viability indicator for environmental bacteria. Cytom. Part A.

[60] Wang M.-S., Wang L.-H., Bekhit A.E.-D.A., Yang J., Hou Z.-P., Wang Y.-Z., Dai Q.-Z., Zeng X.-A. (2018). A review of sublethal effects of pulsed electric field on cells in food processing. J. Food Eng..

[61] García D., Gómez N., Mañas P., Raso J., Pagán R. (2007). Pulsed electric fields cause bacterial envelopes permeabilization depending on the treatment intensity, the treatment medium pH and the microorganism investigated. Int. J. Food Microbiol..

[62] Somolinos M., Mañas P., Condón S., Pagán R., García D. (2008). Recovery of Saccharomyces cerevisiae sublethally injured cells after Pulsed Electric Fields. Int. J. Food Microbiol..

[63] García D., Mañas P., Gomez N., Raso J., Pagán R. (2006). Biosynthetic requirements for the repair of sublethal membrane damage in Escherichia coli cells after pulsed electric fields. J. Appl. Microbiol..

[64] Arroyo C., Somolinos M., Cebrián G., Condón S., Pagán R. (2010). Pulsed electric fields cause sublethal injuries in the outer membrane of Enterobacter sakazakii facilitating the antimicrobial activity of citral. Lett. Appl. Microbiol..

[65] Mishra Y., Chaurasia N., Rai L.C. (2009). AhpC (alkyl hydroperoxide reductase) from Anabaena sp. PCC 7120 protects Escherichia coli from multiple abiotic stresses. Biochem. Biophys. Res. Commun..

[66] Zuo F., Yu R., Khaskheli G.B., Ma H., Chen L., Zeng Z., Mao A., Chen S. (2014). Homologous overexpression of alkyl hydroperoxide reductase subunit C (ahpC) protects Bifidobacterium longum strain NCC2705 from oxidative stress. Res. Microbiol..

[67] Noinaj N., Guillier M., Barnard T.J., Buchanan S.K. (2010). TonB-Dependent Transporters: Regulation, Structure, and Function. Annu. Rev. Microbiol..

[68] Zaborin A., Gerdes S., Holbrook C., Liu D.C., Zaborina O.Y., Alverdy J.C. (2012). Pseudomonas aeruginosa Overrides the Virulence Inducing Effect of Opioids When It Senses an Abundance of Phosphate. PLoS ONE.

[69] Monedero V., Revilla-Guarinos A., Zúñiga M. (2017). Physiological Role of Two-Component Signal Transduction Systems in Food-Associated Lactic Acid Bacteria. Adv. Appl. Microbiol..

[70] De Vrij W., Bulthuis R.A., Van Iwaarden P.R., Konings W.N. (1989). Mechanism of L-glutamate transport in membrane vesicles from Bacillus stearothermophilus. J. Bacteriol..

[71] Rallu F., Gruss A., Ehrlich S.D., Maguin E. (2002). Acid- and multistress-resistant mutants of Lactococcus lactis: Identification of intracellular stress signals. Mol. Microbiol..

[72] Ye Y., Ling N., Gao J., Zhang X., Zhang M., Tong L., Zeng H., Zhang J., Wu Q. (2018). Roles of outer membrane protein W (OmpW) on survival, morphology, and biofilm formation under NaCl stresses in Cronobacter sakazakii. J. Dairy Sci..

[73] Morales E.H., Calderón I.L., Collao B., Gil F., Porwollik S., McClelland M., Saavedra C.P. (2012). Hypochlorous acid and hydrogen peroxide-induced negative regulation of Salmonella enterica serovar Typhimurium ompW by the response regulator ArcA. BMC Microbiol..

[74] Yeats C., Rawlings N.D., Bateman A. (2004). The PepSY domain: A regulator of peptidase activity in the microbial environment?. Trends Biochem. Sci..

[75] Orelle C., Mathieu K., Jault J.-M. (2019). Multidrug ABC transporters in bacteria. Res. Microbiol..

[76] Monaco C., Talà A., Spinosa M.R., Progida C., De Nitto E., Gaballo A., Bruni C.B., Bucci C., Alifano P. (2006). Identification of a Meningococcal l-Glutamate ABC Transporter Operon Essential for Growth in Low-Sodium Environments. Infect. Immun..

[77] Bickle T.A. (2003). Restricting restriction. Mol. Microbiol..

[78] Ziegler C.M., Bremer E., Krämer R. (2010). The BCCT family of carriers: From physiology to crystal structure. Mol. Microbiol..

