# Supplementary material for: Resuscitation of Pulsed Electric Field-Treated Staphylococcus aureus and Pseudomonas putida in a Rich Nutrient Medium
Source: Foods. 2021 Mar 19;10(3):660. doi: 10.3390/foods10030660 (PMC8003612; doi:10.3390/foods10030660)
Supplement: Supplementary file 1 [file foods-10-00660-s001.pdf]

Supplementary material

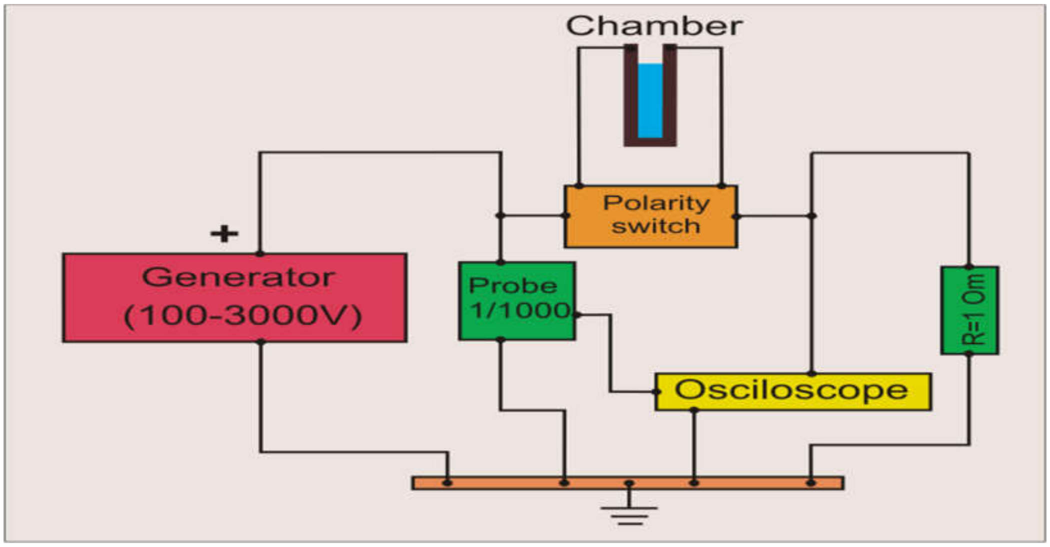

Figure S1. Schematic drawing of the high voltage generator and the electronic circuit

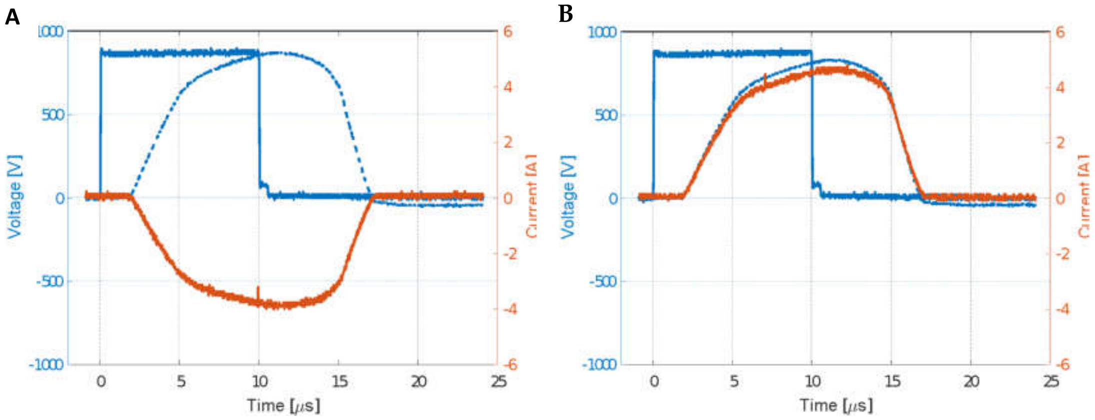

Figure S2. Potential and current time response during a pulse for two cases of opposite polarities (A and B). Voltage input ( ), Voltage output ( ), current output ( ).

A

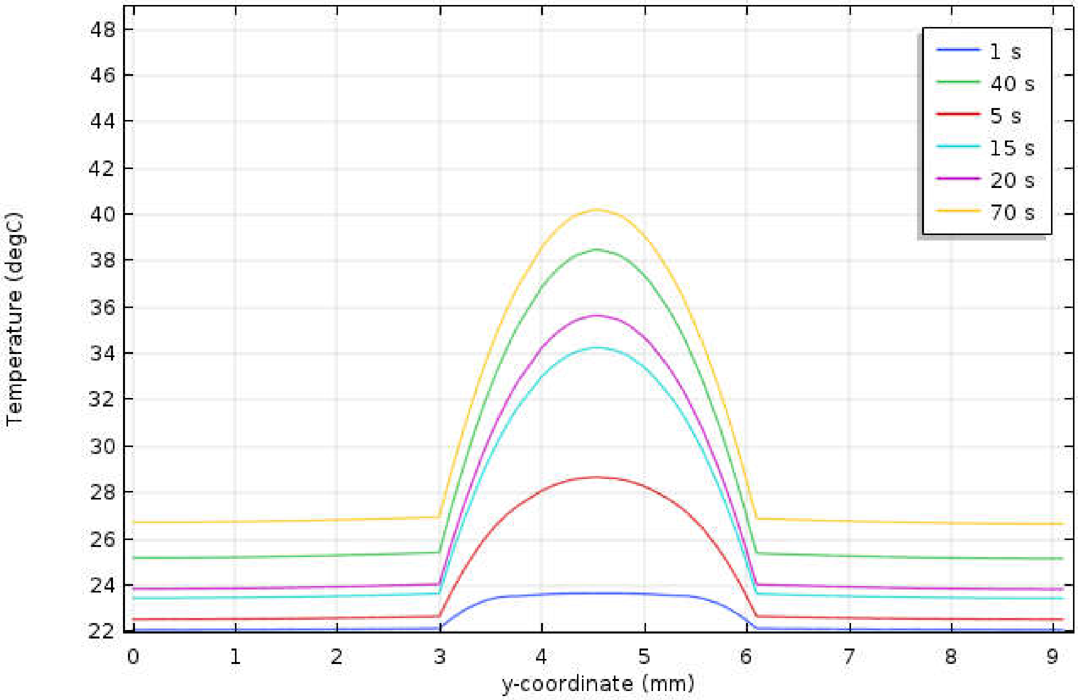

48
46
44
42
40
38
36
34
32
30
28
26
24
22
Temperature (degC)
0
1
2
3
4
5
6
7
8
9
y-coordinate (mm)
1 s
40 s
5 s
15 s
20 s
70 s

B

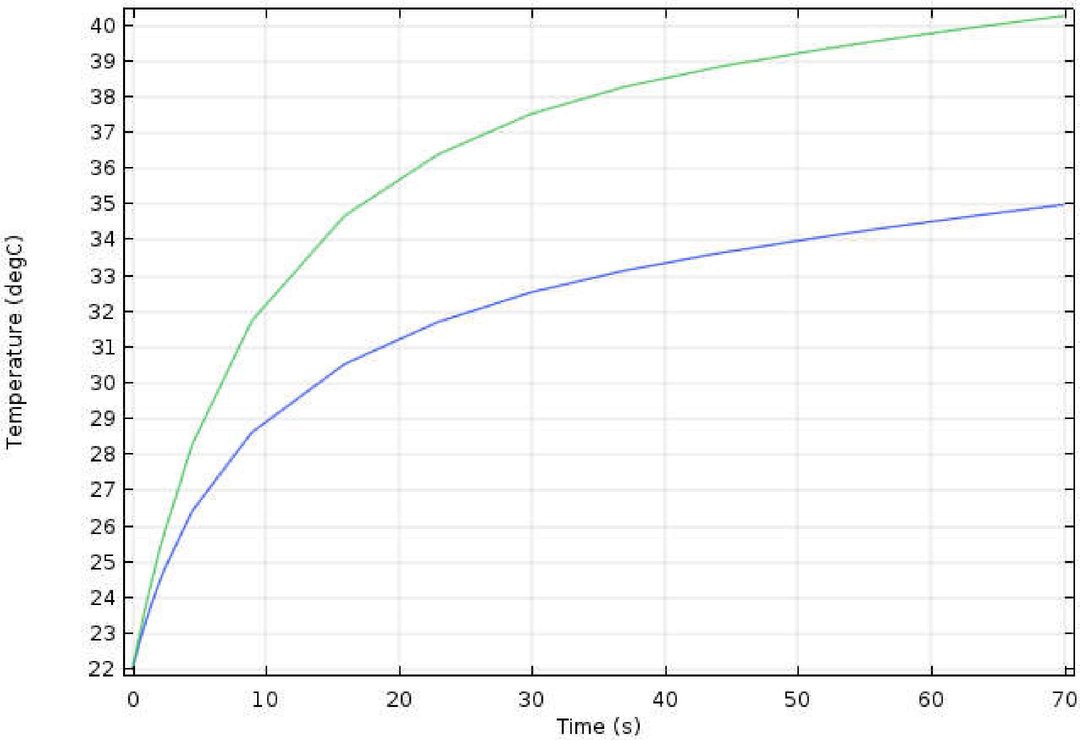

40
39
38
37
36
35
34
33
32
31
30
29
28
27
26
25
24
23
22
Temperature (degC)
0
10
20
30
40
50
60
70
Time (s)

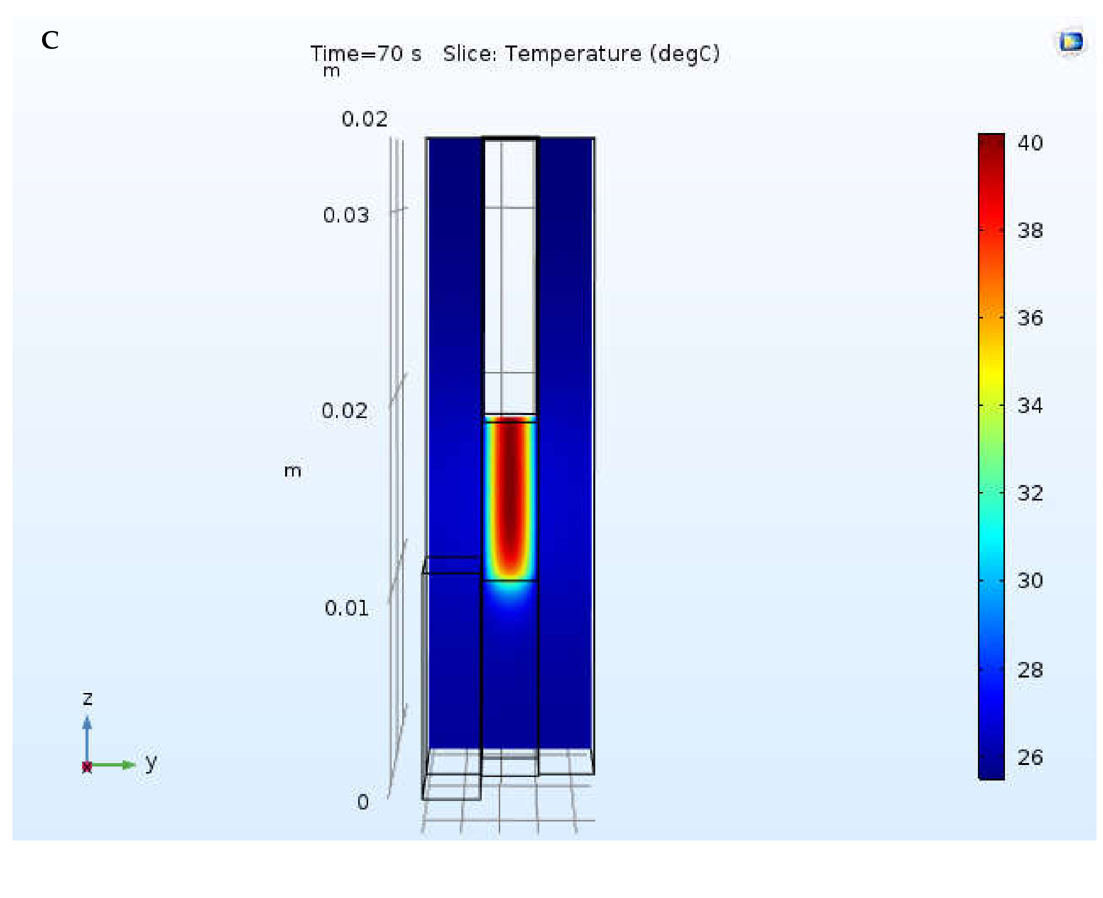

Figure S3. Temperature in the electroporation chamber. Temperature profile at different times during the PEF treatment along the electrodes (A); Maximum temperature ( ) in the electrode sample system, and average temperature ( ) in the electrode sample system (B); Electrode sample system's temperature map after 70 s operation time (C).

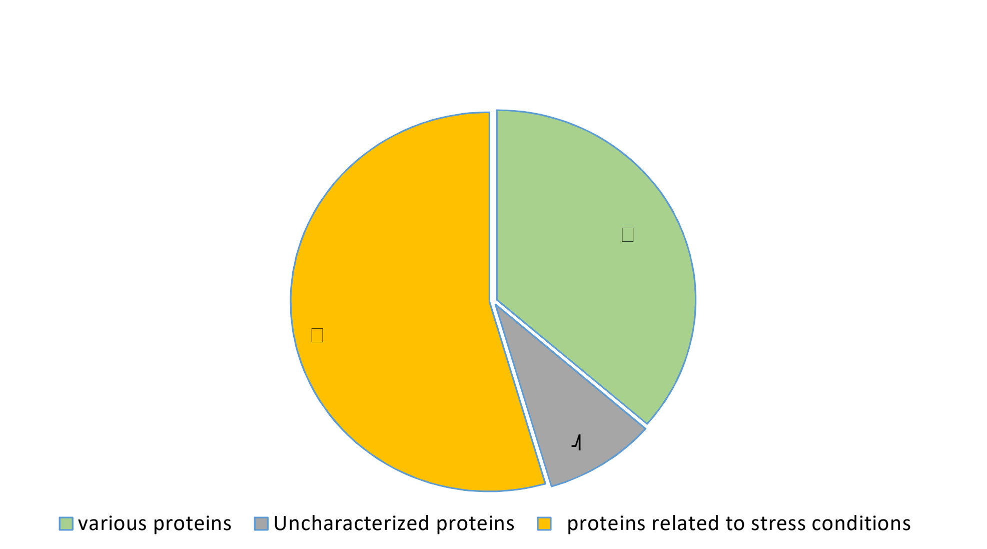

Figure S4. Over-expressed proteins in PEF-treated *P. putida* F1 in BHI, compared to the non-treated sample.
